# Editorial: Insights in thoracic oncology: 2021/2022

**DOI:** 10.3389/fonc.2022.1089540

**Published:** 2022-12-02

**Authors:** Kaushal Parikh, Alfredo Addeo

**Affiliations:** ^1^ Oncology Unit, Mayo Clinic, Rochester, NY, United States; ^2^ Oncology Department, University hospital Geneva, Geneva (CH) Swiss Cancer Centre Leman, Geneva, Switzerland

**Keywords:** NSCL, SCLC, driver mutations, surgery, biomarkers

This Research Topic ‘*Insights in Thoracic Oncology: 2021/2022*’, provide the readers a curated selection of articles to explore the current and future interests in the field of thoracic oncology. These articles encompass multiple disciplines of thoracic oncology and lay out new evidence in diagnosis, prognosis, and treatment of patients with thoracic malignancies. This series features both, high impact original research articles as well as state-of-the-art reviews, suitable for basic, translational and clinical scientists, trainees, and clinicians to stay abreast with this rapidly advancing field. The field is moving forward from genomics to ‘multi’-omics, often using liquid biopsy specimens and minimally invasive techniques. Newer technologies have facilitated measurement and characterization of microRNA (miRNA) in cancer development, treatments, and prognosis and developed into the field of miRNAomics. Dezfuli et al evaluated the expression of miRNAs miR-146a and miR-155 in peripheral blood mononuclear cells. In this case-control study of 33 patients with NSCLC and 30 controls, they discovered significant downregulation of miR146a accompanied by TGF-β elevation. Further work can help develop these are markers for disease progression and outcomes. Arguably, biomarker development for thymic tumors is trailing other thoracic malignancies. Yuan et al elucidated the diagnostic and prognostic role of SOX9 expression in thymic epithelial tumors. They observed that SOX9 was highly expressed in TET cells and correlated with histologic subtypes of thymomas, potentially aiding in diagnosis. They also postulate that SOX9 expression serves as a negative prognostic marker for TETs and was associated with an immunosuppressive tumor microenvironment. The next article in the translational science segment is Johnson et al, wherein provide a comprehensive review on preclinical models and resources to aid research in mesothelioma. They also provide information on mesothelioma biobanks that are available globally to researchers.

For early stage or localized thoracic cancers, surgery with or without perioperative therapy remains the mainstay of treatment. The increased adoption of robotic and other minimally invasive surgical procedures warranted a randomized trial to assess its effectiveness. The ROMAN study by Veronesi et al randomized 83 patients with early-stage NSCLC to robotic-assisted thoracic surgery (RATS) or video-assisted thoracic surgery (VATS) and observed that RATS was not superior to VATS with respect to perioperative outcomes, but it led to a greater degree of hilar and mediastinal nodal assessment compared to VATS. This is the first randomized study comparing two minimally invasive thoracic surgery methods for NSCLC and it sheds light on the need for more validation trials. The second surgical study in this series is a retrospective analysis by Testori et al, where they evaluate the efficacy of intraoperative hypertonic glucose solution administration on persistent air leak after pleurectomy/decortication for pleural mesothelioma^5^. Hypertonic glucose solution is hypothesized to be pro-inflammatory, leading to development of fibrous adhesion between lung and chest wall and resolution of air leak. This case-control study of 71 patients showed that intraoperative hypertonic glucose administration reduced duration of chest tube management after discharge without altering duration of hospitalization or duration of chest tube maintenance during hospitalization. Surgical resection is uncommon in small cell lung cancer, often due to the advanced stage at diagnosis. However, when feasible, surgical resection is the preferred treatment for localized disease and small cell carcinomas are also incidentally diagnosed during surgery at times. Li et al retrospectively analyzed the impact of adjuvant therapy in resected small cell lung cancer. Out of 153 patients included in this single center analysis, adjuvant radiation and adjuvant chemotherapy were associated with improved survival compared to no adjuvant therapy. The benefits adjuvant radiotherapy following chemotherapy were more pronounced for patients with pathologic nodal involvement. This study reiterates the role for surgery and adjuvant therapy in small cell lung cancer.

Non-small cell lung cancer has been the posterchild for targeted therapies and these agents have significantly improved patient outcomes. While osimertinib is the preferred EGFR tyrosine kinase inhibitor (TKI) in the US, several parts of the world have access issues to osimertinib, and earlier generation inhibitors such as afatinib, gefitinib or erlotinib remain standard of care. Passaro et al present a pooled analysis of three single arm phase IIIb studies of afatinib in patients with EGFR mutant NSCLC. Median PFS on afatinib in this analysis was 13.0 months and ORR was 55.0%. Afatinib was found to have no new toxicity signals and showed activity against uncommon EGFR mutations such as G719X, L761X, and S768IX).Patients with previously stable and or treated brain metastases (BM) were included and the efficacy of Afatinib was not affected by the presence of BM. Therefore afatinib could represent a viable frontline option in situations where osimertinib access is limited. EGFR mutations on exon 18 such are uncommon and there is limited evidence on the optimal treatment strategy in these cases. While afatinib is the most widely used TKI in this scenario, Xu et al retrospectively studied the outcome of 82 patients with EGFR exon 18 alterations. They observed no statistically significant difference in PFS between afatinib and the combination of a first generation TKI (erlotinib or gefitinib) with chemotherapy (p=0.709). From discovering and treating new targets, the focus is gradually shifting to overcoming resistance mechanisms and understanding tumor biology of patients with actionable targets. To this effect, Kamga et al studied 74 consecutive patients with EGFR mutant NSCLC and discovered that high circulating plasma levels of sonic hedgehog protein at diagnosis and on treatment with TKIs were associated with worse prognosis and treatment resistance. Another prospective study in this series by Raphael et al concluded that comprehensive genomic profiling of tumor or circulating tumor DNA impacted subsequent treatment in patients with ALK rearranged NSCLC who had disease progression following treatment with a second or third generation ALK TKI. This study further underlines the utility of comprehensive next-generation sequencing upon resistance to targeted therapies. Treatment options post-progression on TKI are limited. If treated with a TKI as frontline therapy, in the absence of targetable resistance mechanisms, treatment is often platinum-based chemotherapy with or without immune checkpoint inhibitors (ICIs). Patients with EGFR or ALK alterations typically respond poorly to ICIs while the responses are somewhat more heterogenous for patients with KRAS, BRAF V600E or MET exon 14 skipping alterations. The articles by Seegobin et al and Wiest et al comprehensively review the role of immune checkpoint inhibitors in treatment of NSCLC with EGFR and other actionable oncogenic driver alterations. In the theme of benefits of ICI in oncogenic-driven NSCLC, Hou et al studied the role of ICI in mice models harboring ATRX-deficient lung cancer cell lines. ATRX is a tumor suppressor gene and ATRX mutations are associated with poor prognosis in multiple cancers. The authors observed that ATRX mutations sensitized lung cancer cell line models to IBI and may serve as a biomarker for benefit with immunotherapy.

The above articles display the tremendous advancements in care of patients with lung cancer. The ultimate goals of cancer care are improving quality of life and prolonging survival. The final two articles in this series focus on these pivotal questions. Guo et al asked the most important question from a patient’s perspective, ‘*How long have I got?’* and retrospectively analyzed 998 patients with metastatic NSCLC to derive an answer. In this real-world analysis, 1-, 2-, and 5-year survival rates were 74%, 49% and 16%, respectively. Their multivariate regression analysis suggested that histopathology, performance score, number of chemotherapy cycles received, and targeted therapy receipt were independently prognostic. However, among patients who survived greater than 3 months, the authors could not identify predictors to differentiate between long-term (>38 months) and short term (<12 months) survivors. This effort highlights our ongoing challenges to accurately predict patient outcomes in lung cancer. Performance status (PS) has been a valuable indicator to guide therapy and prognosis. Performance status, however, is variable and often not an objective assessment. An objective assessment of muscle mass and strength may complement performance status in predicting outcomes. Yang et al prospectively recruited 639 patients with advanced NSCLC and evaluated sarcopenia using CT scan-based skeletal muscle index (SMI) and handgrip strength. They observed that CT-defined sarcopenia alone and in combination with poor handgrip strength were more strongly associated with a poor prognosis than Eastern Cooperative Oncology Group (ECOG) PS≥2 alone (HR 2.0, 95% CI 1.65-2.43; HR 2.00, 95% CI 1.59-2.49). Furthermore, CT-defined sarcopenia, poor handgrip strength and ECOG PS≥2, together defined as severe sarcopenia, was also more strongly associated with poor prognosis compared to ECOG PS≥2 (HR 1.37, 95% CI 1.10-1.73). As we continue to use ECOG PS as a strong prognostic indicator, this prospective study calls for further improvements in our ability to predict patient outcomes.

In conclusion, the articles in this series provide the reader with new and ongoing research in thoracic oncology, review current management strategies and updates, and encourage further contributions in this field to improve lives of patients.

## Author contributions

KP drafted the editorial and for the rest the authors contributed equally. All authors contributed to the article and approved the submitted version.

